# Stability and marginal bone loss in implants placed using piezoelectric osteotomy versus conventional drilling: systematic review and meta-analysis

**DOI:** 10.4317/medoral.24146

**Published:** 2020-11-28

**Authors:** Isabel Godoy-Reina, Gerardo Moreu-Burgos, Maximino González-Jaranay

**Affiliations:** 1 ………; 2 ……..; 3 ….; 4 ….; 5 ….; 6 ….

## Abstract

**Background:**

The main objective of this systematic review was to compare primary and secondary implant stability between placement with piezoelectric osteotomy and conventional drilling, comparing marginal bone losses as a secondary objective.

**Material and Methods:**

An electronic search was conducted using PubMed (MEDLINE), Scopus, and Cochrane Library (Wiley) databases, besides a manual search.

**Results:**

A total of 153 articles were retrieved, 39 from Pubmed, 44 from Scopus, and 70 from the Cochrane Library. After removing duplicates, 112 articles (1 from the manual search) were screened, and 9 were finally selected for qualitative and statistical analyses.

**Conclusions:**

Piezoelectric surgery is a predictable alternative to conventional drilling for dental implant placement. Medium/long-term survival rates and marginal bone losses are similar between piezoelectric osteotomy and conventional drilling, and there is no difference in ISQ values for primary stability. However, implants placed with ultrasound showed a lower decrease in implant stability quotient (ISQ) during the osseointegration period and a higher ISQ value for secondary stability. This study contributes further information on peri-implant bone tissue at 3 and 6 months after implant placement with piezoelectric osteotomy or conventional drilling and provides an updated meta-analysis of comparative studies.

** Key words:**Piezosurgery, piezoelectric surgery, conventional drill, implant site preparation, dental implant, implant stability, marginal bone loss.

## Introduction

The stability of dental implants can be evaluated at any time point by resonance frequency analysis (RFA), a non-invasive procedure, using the implant stability quotient (ISQ) scale (0-100 points) (1). It can also be assessed at implant placement according to the insertion torque (IT), among other techniques. Conventional rotary drilling is the most common approach to bone bed preparation, but other predicTable systems are available. Vercellotti at al. (2) were the first to apply ultrasound in oral surgery using piezoelectric osteotomy (PO), which allows hard tissue to be cut without damaging soft tissues such as oral mucosa, blood vessels, nerves, or Schneider’s membrane (3).

The level of heat generated in the implant bed is a key factor for treatment predictability (4,5), and numerous studies have evaluated the safety of PO for bone bed preparation, comparing the temperature produced by PO versus conventional drilling (CD). One research group (6) reported that the heat generated in the implant bed during PO was not influenced by the degree of pressure exerted but that the irrigation volume was related to the increase in bone cortex temperature. Another study found that bone healing was not affected by the heat generated by CD or PO (7). Preclinical animal research has shown that PO promotes osteogenesis, controls inflammation, and is superior to drilling during the first phases of wound healing (8,9). Some clinical studies have indicated that PO is less invasive than CD, producing peri-implant bone compaction and promoting osteogenesis (10,11), although other authors found no difference between the techniques in the amount of new bone formed during osseointegration (12). Long-term follow-up studies have observed that implants placed with PO have a good survival rate, close to 90-100% (13,14).

The main drawback of PO is considered to be the time needed to prepare the implant bed (15), although one study observed no significant difference in the duration of PO and CD (16). PO has become more widespread due to the lower tissue damage produced.

The main objective of this systematic review was to compare primary and secondary stability in implants between placement with PO and CD, comparing the marginal bone loss (MBL) as a secondary objective.

## Material and Methods

This study was conducted in accordance with PRISMA criteria and guidelines for systematic reviews and meta-analyses (17).

- Focused question

The PICO (population, interventions, comparisons, outcomes) question was: In patients with dental implants, what is the effectiveness of implant bone bed preparation by PO in comparison to CD in terms of primary and secondary stability and MBL?

- Eligibility criteria

Inclusion criteria: Randomized or non-randomized controlled clinical trials; studies comparing clinical or radiological results between implants placed by CD versus PO; studies of primary/secondary stability using resonance frequency analysis (RFA); and studies of MBL during follow-up using radiography.

The study population (P) comprised patients receiving one or more implants by PO (I) and/or CD in any region of the mouth. The outcome (O) was the ISQ value by RFA and/or the MBL by radiographic analysis. Primary and secondary ISQ values were considered as primary response variable and MBL after bone healing as secondary response variable. Data were also gathered on the ultrasound system used, mouth region intervened, follow-up period, and number of implant failures, among other variables.

Exclusion criteria: Studies in which the surgical phase involved additional regenerative treatment; absence of test and control groups; absence of follow-up; observational studies, case series, and reviews.

- Search strategy and data extraction

An electronic search was conducted in PubMed (MEDLINE), Scopus, and Cochrane Library (Wiley) databases until 30 April 2020, with no language or year restrictions, using the following search algorithm: “(piezosurgery OR piezoelectric surgery OR conventional drill) AND (implant site preparation OR dental implant OR implant stability OR marginal bone loss)”. A manual search for eligible studies was also performed in the International Journal of Oral and Maxillofacial Implants, Clinical Oral Implants Research, International Journal of Oral and Maxillofacial Surgery, Journal of Craniofacial Surgery, Journal of Oral implantology, the British Journal of Oral and Maxillofacial Surgery, Clinical Implant Dentistry and Related Research, Implant Dentistry, and Quintessence International.

First, the titles and abstracts of retrieved items were screened by two independent researchers (IGR and GMB), who then applied inclusion and exclusion criteria to the whole text of selected studies. Cohen’s Kappa index was calculated to determine the inter-examiner agreement. Discrepancies were resolved in consultation with a third researcher (MGJ).

Data were gathered on: country; study design; journal; number of implants; number, age, and sex of patients; mouth region intervened; follow-up period; study variables; number of implant failures; and ultrasound device (Table 1). Information was also collected on: implant type, ISQ values, MBL values, and time to definitive restoration (Table 2); ISQ values from implantation (day 0) to 5 months (Table 3); and MBL from 3 to 24 months (Table 4).

**Table 1:**
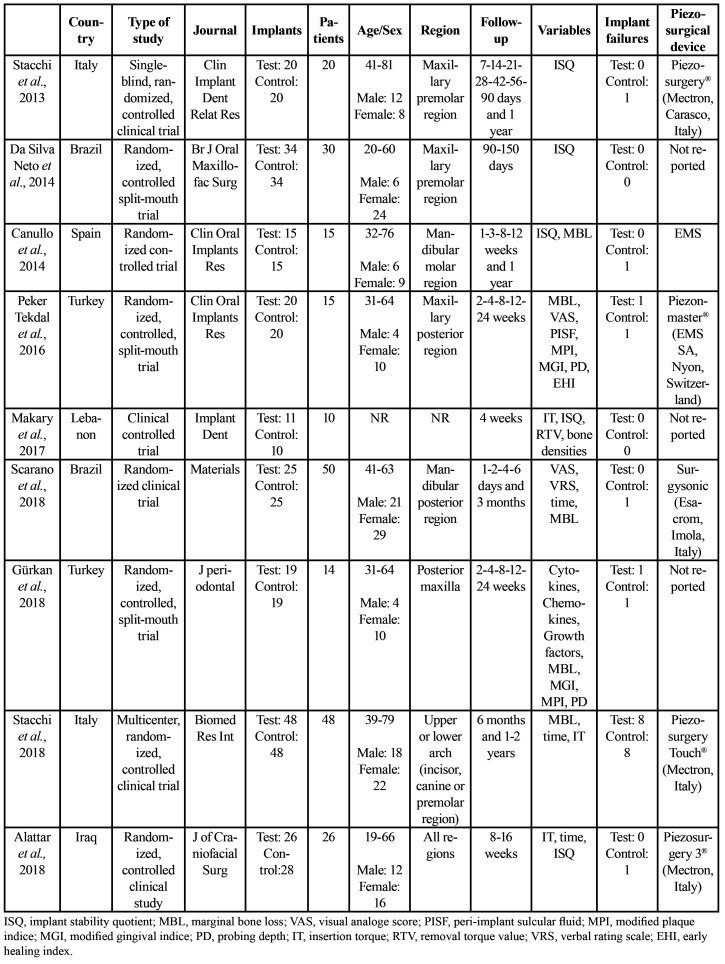
Description of selected studies.

**Table 2:**
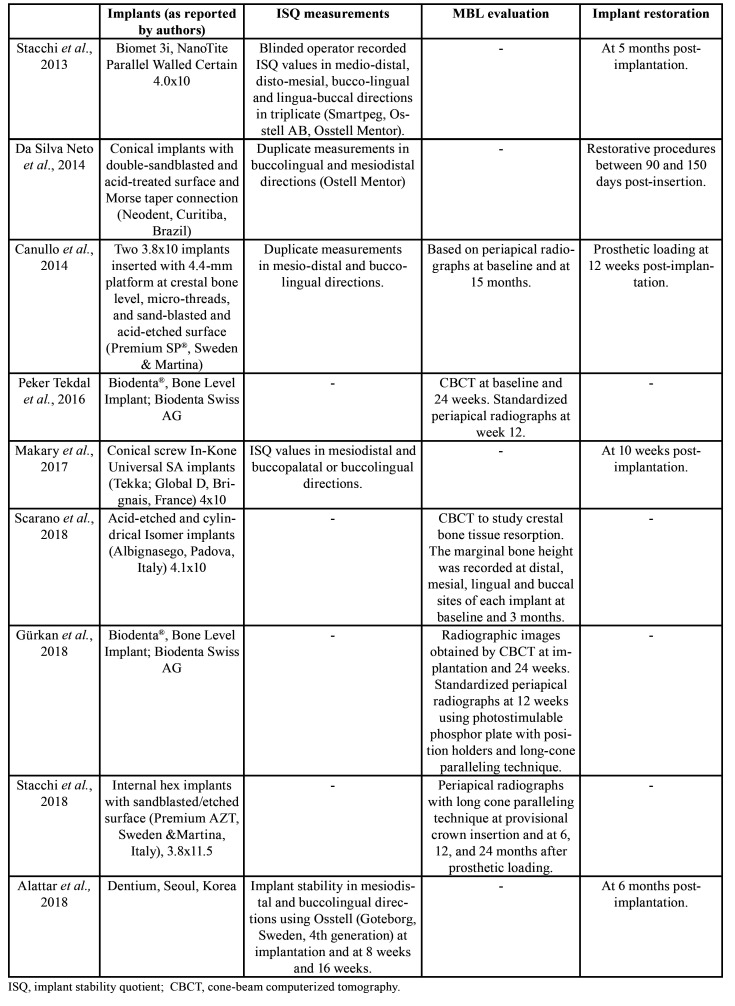
Methodology of selected studies.

**Table 3:**
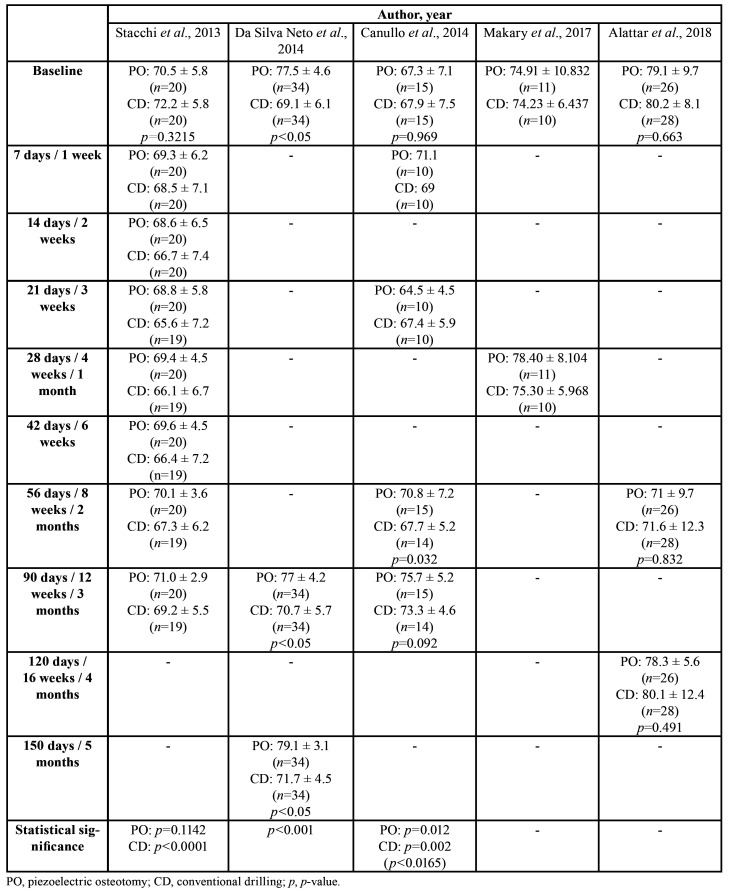
Implant stability results (ISQ values) in different follow-up periods, reported as means with standard deviations.

**Table 4:**
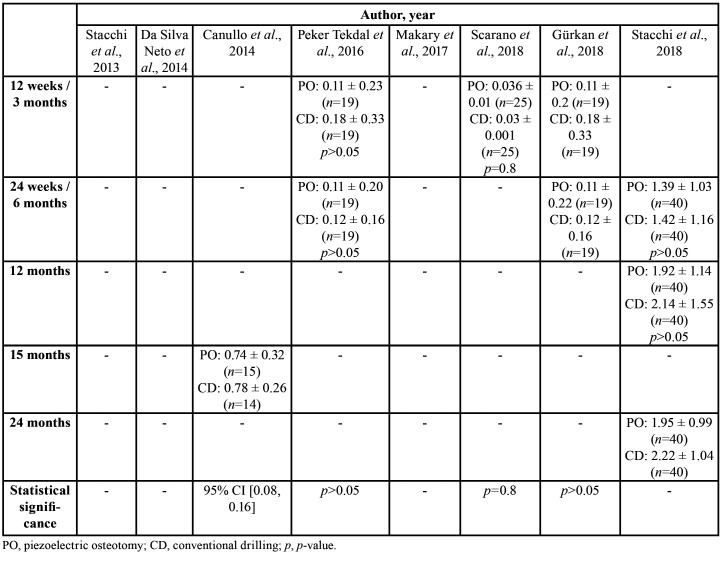
Marginal bone loss (mm) in different follow-up periods, reported as means with standard deviations.

- Risk of bias

The risk of bias in each study was evaluated independently by two researchers (IGR and GMB) in accordance with the Cochrane collaboration manual and instrument for systematic reviews of intervention studies (RevMan, version 5.3). They examined the random sequence generation, allocation concealment, blinding of participants/personnel, blinding of outcome assessment, incomplete outcome data, selective reporting, and other sources of bias, classifying studies into three groups: “low-risk bias”, when there was little likelihood that bias weakened results; “high-risk bias”, when a potential bias reduced the reliability of results; or “unclear-risk bias, when there was a lack of detail on potential sources of bias.

- Statistical Analysis

Program Review Manager (RevMan, The Cochrane Collaboration, Copenhagen, Denmark; 2014) version 5.3 was used for the meta-analysis of comparisons in ISQ and MBL values between PO and CD groups, considering the implant as statistical unit. The standardized mean difference (SMD) was calculated for continuous data with random-effect models and 95% confidence interval (CI). *P* <0.05 was considered statistically significant. Heterogeneity was estimated based on the Cochrane Q-test, considering *p*<0.10 to be significant, and on the I2 index, classifying I2=25 % as low, I2=50 % as moderate, I2=75 % as high. It was not possible to analyze the publication bias because there were fewer than 10 studies in the meta-analysis (18). Between-group comparisons were conducted of primary stability at baseline, secondary stability at 2 and 3 months, and MBL at 3 and 6 months.

## Results

- Study selection

**Figure 1:**
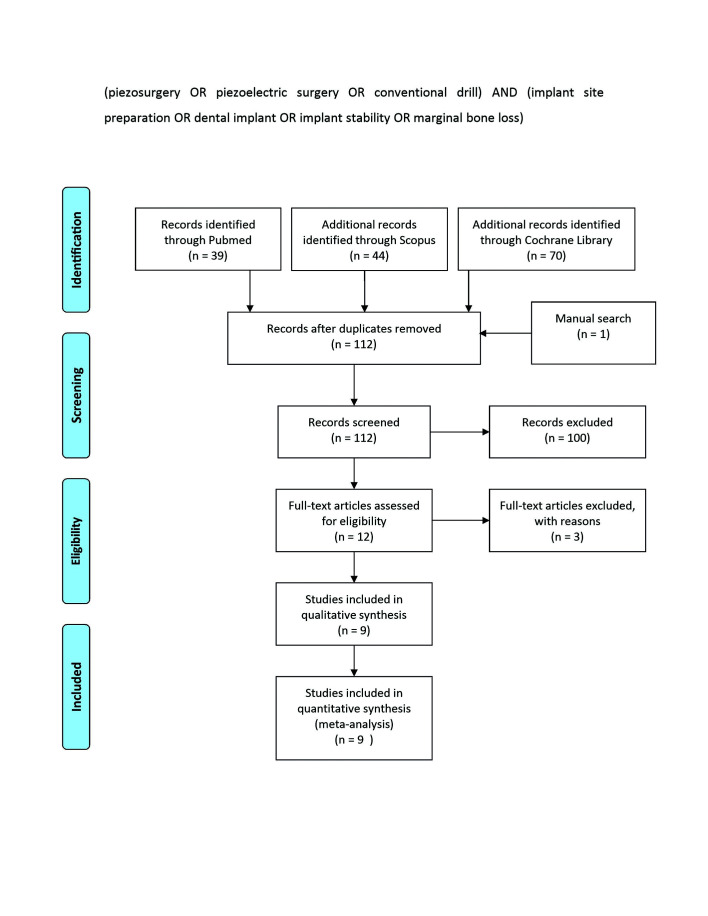
Flow chart of search process.

The search strategy yielded a total of 153 articles: 39 from PubMed, 44 from Scopus, and 70 from the Cochrane Library. After removing duplicates, 112 articles were selected for subsequent screening, including one retrieved in the manual search (18). Nine articles were finally selected for qualitative and statistical analyses (Fig. 1). Cohen’s Kappa index for inter-examiner agreement was K=0.80, considered very good (0.80-1.00).

- Study characteristics

Among the nine studies in the review, eight are randomized controlled trials (RCTs) (19-26) and one is a non-randomized controlled clinical trial (27).

The studies included a total of 228 patients aged between 20 and 81 years, 83 males and 128 females, although data on age and sex were not available in one of the studies (27). Out of the total of 437 implants placed, the preparation was performed by PO in 218 and by CD in 219. The ISQ response variable was evaluated in five studies (19-21,23,27) and the MBL response variable in five studies (20,22,24-26).

- Risk of bias

**Figure 2:**
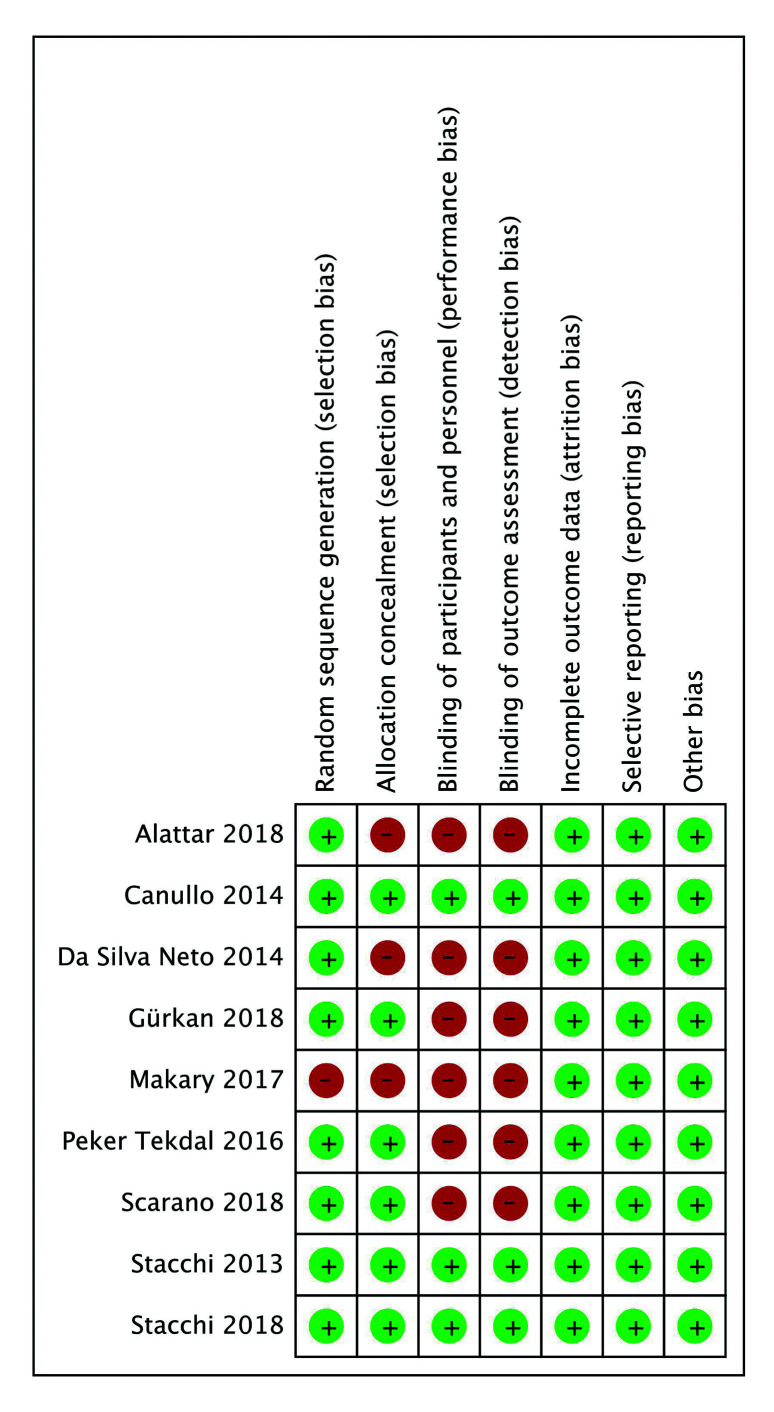
Risk of bias assessment.

Selection bias was found in three studies, observing lack of allocation concealment in two (21,23) combined with non-randomized sequence generation in the third (27). Performance and detection bias was observed in six studies due to the non-blinding of participants, staff, or evaluators (21-24,26,27). Only three studies were classified as having a low risk of bias (19,20,25) (Fig. 2).

- Qualitative synthesis

Results for response variables are exhibited in Tables 3 and 4. The mouth region intervened was described by all studies except for that by Makary *et al*. (27). Maxillary premolars were studied by Stacchi *et al*. (19) and da Silva Neto *et al*. (21), mandibular molars by Canullo *et al*. (20) and Scarano *et al*. (24), the posterior maxillary region by Peker Tekdal *et al*. (22) and Gürkan *et al*. (26), the region from incisors to maxillary/mandibular premolars by Stacchi *et al*. (25), and all regions by Alattar *et al*. (23). Three studies had a split-mouth design (21,22,26), and PO- and CD-prepared implants were placed in adjacent teeth in another (20). The follow-up period ranged from one day post-intervention (24) to two years (25). There were 10 implant failures in the PO group and 14 in CD group. Table 1 lists all of these results.

ISQ values were compared between the time of placement and subsequent time points by Stacchi *et al*. (19), da Silva Neto *et al*. (21), Canullo *et al*. (20), Makary *et al*. (27), and Alattar *et al*. (23). Canullo *et al*. (20) found no statistically significant difference between PO and CD in stability at baseline (67.3±7.1 vs. 67.9±7.5, respectively, *p*=0.969) or at 12 weeks (75.7±5.2 vs. 73.3±4.6, *p*=0.092), but observed a significantly higher ISQ value in the PO group at 8 weeks (70.8±7.2 vs. 67.7±5.2, *p*=0.032). Stacchi *et al*. (19) also found no statistically significant difference in primary stability (70.5±5.8 vs. 72.2±5.8, respectively *p*=0.3215) but reported higher ISQ values for PO versus CD during the final stages of osseointegration, mainly from day 14 to 42 (*p*<0.0001). In the same line, no significant between-group differences in ISQ values were found by Makary *et al*. (27) at placement (74.91±10.832 vs. 74.23±6.437) or 4 weeks (78.40±8.104 vs. 75.30±5.968) or by Alattar *et al*. (23) at placement (PS:79.1±9.7, CD:80.2±8.1, *p*=0.663), 8 weeks (PS:71±9.7, CD:71.6 ±12.3, *p*=0.832), or 16 weeks (PS:78.3±5.6, CD:80.1±12.4, *p*=0.491). However, da Silva Neto *et al*. (21) described significantly higher ISQ levels with PO at placement (77.5±4.6 vs. 69.1±6.1, *p*<0.05), at 90 days (77±4.2 vs. 70.7+-5.7, *p*<0.05), and at 150 days (79.1±3.1 vs. 71.1±4.5, *p*<0.05).

With regard to MBL results, Canullo *et al*. (20) observed no statistically significant differences between PO and CD at 15 months (0.74±0.32 vs. 0.78±0.26, 95% CI [0.08, 0.16]). Peker Tekdal *et al*. (22) also found no significant between-group difference in MBL at 12 weeks as measured by periapical X-ray (0.11±0.23 vs. 0.18±0.33, *p*>0.05) or at 24 weeks as determined by cone beam computed tomography (0.11±0.20 vs. 0.12±0.16, *p*>0.05); highly similar MBL results were described by Gürkan *et al*. (26) suggesting a possible overlap in their study populations. Likewise, no statistically significant between-group difference in MBL was observed by Scarano *et al*. (24) at 3 months (0.036±0.01 vs. 0.03±0.001, *p*=0.8), or by Stacchi *et al*. (25) at 6 months (1.39±1.03 vs. 1.42±1.16, *p*>0.05) or 12 months (1.92±1.14 vs. 2.14±1.55, *p*>0.05).

- Quantitative synthesis

**Figure 3:**
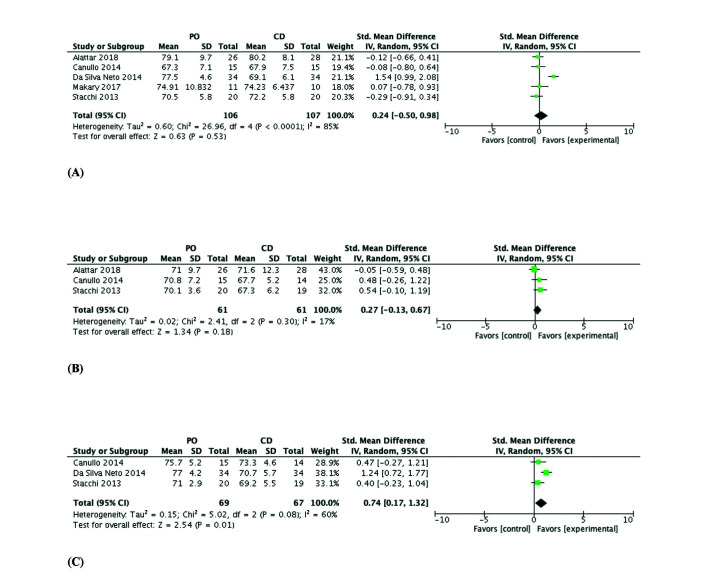
Forest plot (random-effects model). Implant secondary stability, ISQ at baseline/time 0 (A), at 2 months (B) and at 3 months (C). PO, piezoelectric osteotomy; CD, conventional drilling; CI, confidence interval; IV, inverse variance.

Meta-analysis results showed no significant differences in primary stability between implants placed by PO or CD (SMD of 0.24; 95%CI: 0.50, 0.98; *p*=0.531), with a high heterogeneity (T2=0.60; X2=26.96; df=4; *p*<0.0001; I2=85%) (Fig. 3). There was no significant between-group difference in secondary stability at 2 months (0.27 SMD; 95% CI: 0.13, 0.67; *p*=0.18), with a low heterogeneity (T2=0.02; X2=2.41; df=2; *p*=0.30; I2=17%) (Fig. 3); however, a significantly higher stability was observed with PO versus CD at three months (0.74 SMD; 95% CI: 0.17, 1.32; *p*=0.01), with a moderate heterogeneity (T2=0.15; X2=5.02; df=2; *p*=0.008; I2=60%) (Fig. 3).

**Figure 4:**
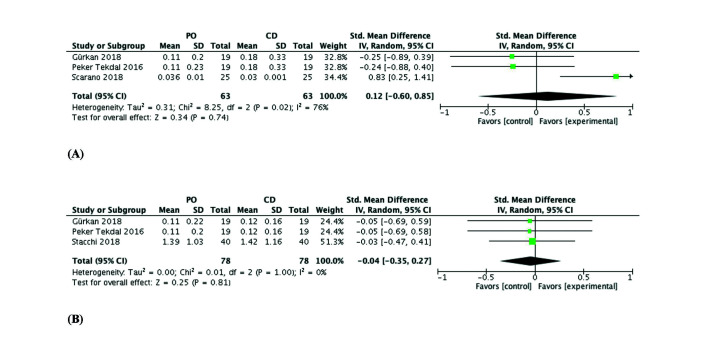
Forest plot (random-effects model). Marginal bone loss (MBL) at 3 months (A) and at 6 months (B). PO, piezoelectric osteotomy; CD, conventional drilling; CI, confidence interval; IV, inverse variance.

No significant between-group difference in MBL was observed at 3 months (0.12 SMD; 95% CI -0.60, 0.85; *p*=0.74), with a high heterogeneity (T2=0.31; X2=8.25; df=2; *p*=0.02; I2=76%) (Fig. 4), or at 6 months (-0.04 SMD; 95% CI -0.35, 0.27; *p*=0.81), with a low heterogeneity that was attribuTable to the similar results published by two of the compared studies (T2=0.00; X2=0.01; df=2; *p*=1.00; I2=0%) (Fig. 4).

## Discussion

This updated review on the comparative merits of PO and CD for implant bed preparation contributes new evidence on the primary and secondary stability (ISQ) and MBL values obtained with each approach. Only nine studies met the eligibility criteria for inclusion, and only one of these was found to be at low risk of bias, highlighting the need for further good-quality studies, especially on long-term outcomes. Implant stability can be measured by RFA in a non-invasive manner at any stage of osseointegration (1) and by the IT at implant placement. However, the present meta-analysis only includes ISQ values, because these provide information on implant stability not only at the time of implantation but also at subsequent stages of osseointegration. With regard to IT values, no between-group difference was found by Peker Tekdal *et al*. (22), Alattar. *et al*. (23) or Makary *et al*. (27). Besides reporting a direct relationship between IT value and bone density, Makary *et al*. (27) also found no between-group difference in reverse torque levels measured using a dynometric wrench at 4 weeks after implant placement. In an *in vitro* study of bones of different densities, Sagheb *et al*. (28) observed that RFA results did not differ between PO and CD and that reverse torque values were related to bone density, being highest in implants placed in mixed/cortical bone by PO, whereas another in-vitro study obtained the highest ISQ value for implants placed in cancellous bone, also using PO (29). In addition, no difference in RFA-measured primary stability was observed between the techniques in an animal study by Bengazi *et al*. (30). In the present review, only da Silva Neto *et al*. (21) demonstrated significantly superior primary and secondary stability (ISQ values) with PO versus CD, while another two studies described higher values for PO-prepared implants during the final stages of osseointegration (19,20). Two of the reviewed studies used a mixed technique (drilling and PO), which may affect the comparative results obtained in the meta-analysis (20,23). Only one study performed immediate loading in both groups, observing no difference between them in survival rates (25).

It was recently reported that implant preparation with piezoelectric surgery favors osteoblast viability, thereby improving bone healing (31). It was also found to reduce the destructive inflammatory response of bone during osseointegration, and it may therefore be less traumatic at molecular level in comparison to drilling, although this was not reflected in bone loss values (22). These findings may explain the lower pain and inflammation reported by Scarano *et al*. (24) with PO versus CD, although other studies found no between-group differences in molecular biomarkers (cytokines, chemokines, and growth factors) or bone repair mechanisms (osteoprotegerin, RANK-L, osteocalcin, caspase-3 proteins) (26,32).

This meta-analysis found no difference in MBL between implants prepared by PO and CD at implant placement or at 3 or 6 months. Further studies with longer follow-ups are needed to improve knowledge of the response of peri-implant tissues over the longer term. Implants placed by PO have been associated with high survival rates, as confirmed in the latest follow-up study (14). Piezoelectric surgery is a predicTable alternative to drilling, although it requires more time (23,24). Some authors combined initial CD with subsequent bone bed preparation using ultrasound inserts, and the time required was closer to that needed for CD alone (20,23).

In the reviewed studies, no differences in primary stability (ISQ values) were observed between implants prepared with PO versus CD, but the stability obtained with PO was superior at 3 months, possibly due to more rapid bone remodeling or healing, with a lesser reduction in osseointegration. Further comparative studies are required to evaluate the effects of reducing the interval before implant loading.

Very few studies have compared the MBL between PO and CD (33-35), and only Atieh *et al*. (15) compared this variable after different follow-up periods. The present study provides further information on the effects of implant placement by piezoelectric surgery or CD on peri-implant bone tissue at 3 and 6 months, including new original studies in an updated meta-analysis (23-27).

## Conclusions

Piezoelectric surgery is a predicTable alternative to conventional drilling for implant bed preparation and obtains similar primary and secondary stability (ISQ values) and MBL values, at least over the short/medium term (six months), although the reduction in stability during the osseointegration period appears to be lesser with ultrasound. Medium/long-term survival rates and marginal bone losses are similar between piezoelectric osteotomy and conventional drilling, which do not differ in ISQ values for primary stability. However, implants placed with ultrasound show a lower decrease in ISQ during the osseointegration period and a higher ISQ value for secondary stability. Further good-quality research is required to compare stability and bone loss values between these techniques over the longer term and to examine the safety of ultrasound in immediate loading protocols.
